# Plasma levels of hypoxia-regulated factors in patients with age-related macular degeneration

**DOI:** 10.1007/s00417-017-3846-z

**Published:** 2017-11-25

**Authors:** Zygoula Ioanna, Schori Christian, Grimm Christian, Barthelmes Daniel

**Affiliations:** 1 Department of Ophthalmology, University Hospital Zurich, Frauenklinikstrasse 24, 8091 Zurich, Switzerland; 2 Lab for Retinal Cell Biology, Department of Ophthalmology, Zurich Centre for Integrative Human Physiology (ZIHP), and Neuroscience Centre (ZNZ), University of Zurich, Zurich, Switzerland

**Keywords:** Age-related macular degeneration, Vascular endothelial growth factor, Cytokines, Plasma

## Abstract

**Purpose:**

Various hypoxia-related proteins are differentially expressed in the retina and secreted to the vitreous and/or aqueous humor of patients affected by dry or neovascular age-related macular degeneration (nAMD). To determine whether these conditions alter concentrations of cytokines also in the systemic circulation, we measured plasma levels of six hypoxia-related proteins.

**Methods:**

Plasma was prepared from EDTA blood that was collected from patients affected by dry AMD (*n* = 5), nAMD (*n* = 11), proliferative diabetic retinopathy (PDR; *n* = 9), and patients with an epiretinal membrane (ERM; *n* = 11). ERM samples served as negative controls, PDR samples as positive controls. Protein concentrations of vascular endothelial growth factor (VEGF), erythropoietin (EPO), angiopoietin-like 4 (ANGPTL4), placental growth factor (PlGF), tumor necrosis factor alpha (TNF-α), and pigment epithelium-derived factor (PEDF) were determined by enzyme-linked immunosorbent assay (ELISA).

**Results:**

The concentration of PlGF was significantly increased in plasma of patients affected by nAMD. Although no statistically significant differences were found for EPO, ANGPTL4, PlGF, TNF-α, and PEDF, the mean concentration of VEGF was lowest in the nAMD group. Plasma concentrations of the six factors did not correlate with gender or age of patients.

**Conclusions:**

nAMD may increase plasma concentrations of PlGF, making it a candidate as a biomarker for the neovascular form of AMD. Other factors, however, were not differentially regulated, suggesting that their systemic concentrations are not generally increased in hypoxia-related retinal diseases.

## Introduction

Age-related macular degeneration (AMD) is a leading cause of irreversible and progressive vision loss among the elderly in the Western world [1–3]. While deterioration of vision in dry AMD (geographic atrophy) occurs slowly, vision loss in neovascular AMD (nAMD) often happens within a few months [4, 5]. The severity and socio-economic impact of AMD combined with its increasing incidence makes an efficient treatment an urgent medical need. Several compounds that target vascular endothelial growth factor (VEGF) have been developed that demonstrate unprecedented results in randomized clinical trials preventing vision loss in the majority of patients with nAMD [6–9]. However, to date, no therapy has been approved for dry AMD.

Understanding the pathogenesis of dry AMD, i.e., the processes leading to the formation of geographic atrophy, is critical for the development of efficient therapies. Apart from known risk factors such as advanced age, cigarette smoke, high body mass index, and genetic variants [10–12], inflammation and oxidative damage have been implicated in disease induction and/or progression [13]. Recently, it was hypothesized that reduced tissue oxygenation (hypoxia) may be another contributing factor not only for the pathogenesis of nAMD but also of dry AMD [14, 15]. Reduced choroidal blood flow and tissue changes in the aging eye may reduce oxygen delivery to the retinal pigment epithelium (RPE) and the outer layer of the neuronal retina, potentially inducing mild but chronic hypoxia in photoreceptor cells and RPE. This may lead to the activation of hypoxia-inducible transcription factors (HIFs) [16, 17] and consequently to the induction of HIF target genes including VEGF [18, 19], a main factor for the development of choroidal neovascularization in nAMD [20–23]. In addition to VEGF, hypoxia increases expression of several additional factors such as angiopoietin like 4 (ANGPTL4), placental growth factor (PlGF), pigment epithelium derived factor (PEDF), tumor necrosis factor (TNF-α), erythropoietin (EPO), and others that have been implicated in angiogenesis, neovascularization, and/or inflammation [24–29]. To address whether patients suffering from retinal disease with a hypoxic component also show systemic changes of these hypoxia-regulated proteins, we studied plasma samples from patients with nAMD, dry AMD and patients with proliferative diabetic retinopathy (PDR). The non-hypoxic control group comprised of samples from patients with epiretinal membranes (ERM).

## Materials and methods

### Patients, collection of plasma, and ELISA

This non-interventional, single-center study at the Department of Ophthalmology at the University Hospital Zurich enrolled 36 participants and was approved by the human ethics committee of the Canton of Zurich, Switzerland. All study subjects were recruited among patients who were regularly scheduled for cataract surgery or vitrectomy. Informed consent was obtained from all participants prior to participation. The study adheres to the tenets of the Declaration of Helsinki. All participants underwent ophthalmologic examination 1 day before and 1 day after surgery as well as follow-up examinations after 1 month. The blood samples were collected by venous puncture prior to surgery, i.e., at a time when no additional oxygenation or medication was administered. At the time of taking blood samples, patients had been fasting for at least 6 h.

Exclusion criteria were: glaucoma, intraocular surgery within the last 6 months, ocular medications other than lubricants, intraocular inflammation, non-proliferative diabetic retinopathy, myopia of more than 6 diopters spherical equivalent, any other ocular vascular disease, previous retinal detachment, previous vitrectomy, retinal degenerative disease, and presence of any other retinal condition potentially affecting either function or oxygenation of the retina other than nAMD, dry AMD, or proliferative diabetic retinopathy (PDR). Patients with PDR or nAMD who received anti-VEGF treatments must have had a minimum interval of 2 weeks between sample collection and last intravitreal injection of an anti-VEGF drug.

Patients were assessed clinically during routine clinics and underwent slit-lamp examination, visual acuity testing, measurement of intraocular pressure, and optical coherence imaging.

Patients with PDR are known to have a strong vascular response that involves activation of HIF transcription factors due to local hypoxia caused by reduced retinal perfusion and microvascular complications [30–35]. These samples served as a “positive control” for a hypoxic ocular response. All participants were ophthalmological patients referred to undergo intraocular surgery, thus we selected patients with ERM to serve as “negative control” because they are not known to have any hypoxia-related changes in the retina.

Based on available literature, we selected VEGF, EPO, ANGPTL4, PlGF, TNF-α, and PEDF as hypoxia-related factors [24, 25, 27, 36–39] to be tested in plasma as follows: Blood was collected in EDTA tubes and kept on ice until use. Plasma was prepared by centrifugation of the EDTA blood at 1300 ×* g* for 15 min within 2 h after collection. Aliquots were prepared and stored in liquid nitrogen until analysis. Plasma concentrations of the six factors were determined by ELISA using the DuoSet (ANGPTL4 and PEDF) or the Quantikine format (VEGF, EPO, PlGF, TNF-α) according to manufacturer’s instructions (R & D Systems Inc., Minneapolis, Minnesota, USA). The minimal detectable dose (MDD) was defined as the value received by addition of two standard deviations to the mean optical density value of zero standard measurements. For the different factors the MDD was as follows: VEGF: 9 pg/ml; EPO: 6 mIU/ml; PlGF: 0.14 pg/ml; TNF-α: 0.12 pg/ml; ANGPTL4: 0.79 ng/ml; PEDF: 2.21 ng/ml.

### Statistical analyses

Quantification of ELISA data was done by GraphPad Prism version 6.0f for Mac OS X (GraphPad Software, La Jolla, CA, USA) using Sigmoidal 4PL fit with 1/y^2^ correction for heteroscedasticity. Numbers were expressed as median (interquartile range (IQR)) Differences among groups were analyzed by one way analysis of variance (ANOVA), followed by Kruskal–Wallis rank sum test with Benjamini–Hochberg post-test for individual comparisons between groups. This statistical analysis was performed by R (Version 3.2.3) and R Studio (Version 0.99.887; R Core Team (2015), Vienna, Austria) with Ggplot2 (H. Wickham, 2009), dunn.test (A. Dinno, 2016) packages.

## Results

### Patients/demographics

Blood plasma samples were collected from 36 patients that were diagnosed with nAMD (11 patients), dry AMD (five patients) or PDR (nine patients). Eleven patients with an ERM served as controls. The mean age of all patients was 72 ± 14 years. Detailed demographic data are shown below in Table 1.

**Table 1:** Patient groups and demographic data

Disease group	No. of patients	Age (years); mean ± SE	Gender	
			Male	Female
Dry AMD	5	80 ± 7	1	4
nAMD	11	80 ± 5	4	7
PDR	9	59 ± 2	4	5
ERM	11	71 ± 4	5	6

All patients with nAMD received intravitreal injections of anti-VEGF drugs. Cataract surgeries were scheduled between two consecutive injections. Only three of the nine patients with PDR received intravitreal ranibizumab as only in those three a macular edema was diagnosed. All of the patients had undergone peripheral laser photocoagulation. Intraocular pressure was within normal ranges in all patient groups. Clinical information is detailed in Table 2.

**Table 2:** Clinical data

Disease group	Visual acuity		Intraocular pressure (mean)	Anti-VEGF therapy	Peripheral laser coagulation
	Min	Max			
Dry AMD	20/60	20/40	14 mmHg	–	
nAMD	Hand movements	20/60	14 mmHg	5/11 ranibizumab and aflibercept	
				5/11 ranibizumab only	
				1/11 aflibercept only	
PDR	Counting fingers	20/100	15 mmHg	3/9 ranibizumab only	9/9
ERM	20/200	20/30	16 mmHg	–	

### Plasma levels of hypoxia-related factors

**Fig. 1:**
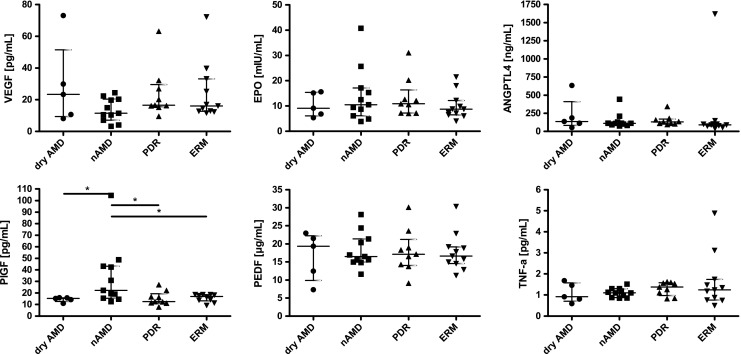
Concentrations of factors in plasma of patients. Shown are individual data points, as well as median (IQR). *N* = 5 (dry AMD), *N* = 11 (nAMD), *N* = 9 (PDR), *N* = 11 (ERM). *: *P* < 0.05

Nearly all measured values were in the normal range reported for the factors in human plasma [40–48]. Plasma levels of PlGF were significantly higher in nAMD patients than in all other patient groups (Fig. 1).

Although all other factors analyzed were statistically similarly expressed in all groups, VEGF showed a tendency towards lower levels in patients suffering from nAMD. As stated, all of these patients underwent intraocular anti-VEGF therapy 2–4 weeks before collection of plasma suggesting that the local treatment affected systemic VEGF levels. Mean levels of EPO were slightly elevated in patients of the nAMD and PDR groups (Fig. 1). Both nAMD and PDR have an established hypoxic component. Values outside IQR were from different patients, except for VEGF and EPO in the dry AMD group and PEDF and EPO in the ERM group. Notably, the two patients with VEGF values above average in the dry AMD group also had above-average levels for EPO, PEDF, and TNF. One of the two patients had also above-average levels of PlGF in addition. No correlation between plasma levels of the factors to sex or age of patients was found.

## Discussion

The aim of our study was to evaluate whether plasma levels of hypoxia-related factors implicated in pathologic angiogenesis such as in nAMD, dry AMD, or PDR are significantly altered. To our knowledge, this is the first study simultaneously comparing plasma levels of factors implicated in hypoxia-related tissue responses in four different patient groups.

Although VEGF is considered to be the most important angiogenic factor for the development of retinal and choroidal neovascularization [49], additional factors such as EPO [50], ANGPTL4 [24], PlGF [51], and others may also contribute to disease development. TNF-α for example has been found in the ischemic retina [52], which implicates it in the response to hypoxia and in retinal angiogenesis, even though TNF-α has mostly been connected to inflammatory processes. PEDF is considered as an anti-angiogenic factor counteracting VEGF [53]. Misregulation in hypoxia may result in an imbalance between PEDF and VEGF potentially contributing to retinal neovascularization [54, 55]. Here we show that only PlGF was significantly increased in the plasma of nAMD patients whereas levels of VEGF, EPO, PEDF, ANGPTL4, and TNF-α did not significantly vary across patient groups. Although it has been reported that PlGF contributes to choroidal neovascularization [25], PlGF has not yet been determined in the circulation of AMD patients. We found that the plasma concentration of PlGF was significantly elevated in nAMD and differed from the other patient groups, as shown in Fig. 1. However, only about 50% of the nAMD patients presented plasma levels that were above the median of the control group. Clearly, a more detailed investigation with an increased cohort size and a careful correlation of PlGF levels with the severity of disease, status of therapy and response efficacy to anti-VEGF treatment is needed to identify the reason for this variability. At this point, it should be mentioned that apart from angiogenesis, PlGF has been associated with other systemic diseases including atherosclerosis [56], hypertension [57], and coronary artery disease [58]. Similarly, cardiovascular disease and hypertension have also been associated with nAMD [59]. On the other hand, despite the fact of a relatively high prevalence of these diseases in the elderly, they are not exclusive for a particular eye condition; thus it is likely that these effects would be leveled out among the different patient groups. However, since the present study was conducted without taking into consideration any systemic diseases, a possible influence of any systemic disease on the plasma levels of the studied factors cannot be neglected. However, PlGF is an interesting and promising candidate for further studies.

The lowest median plasma concentration of VEGF was unexpectedly found in nAMD patients (Fig. 1) where high intraocular levels of VEGF are causative for the neovascularization that characterizes the disease. The low plasma levels of VEGF were possibly related to the anti-VEGF treatment that all nAMD patients had received 2–4 weeks prior to sample collection. It is interesting to note that median VEGF levels in the dry AMD group tended to be elevated when compared to controls, implying a potential contribution of VEGF and/or hypoxia to the development or progression of this disease. Although earlier reports show elevated VEGF levels in plasma of PDR patients [60–62], VEGF was not above control in our PDR patient group (Fig. 1). All our PDR patients had received panretinal laser coagulation and three of them at least once an anti-VEGF therapy prior to surgery, potentially explaining this difference. Alternatively, the discrepancy may be based on the generally good control of blood sugar levels in Swiss diabetics [63]: it has been reported that plasma levels of VEGF were elevated in diabetics with poor blood sugar control but dropped to the normal range after patients had achieved better control [64]. In addition, the studies reporting higher VEGF levels were conducted in non-Caucasians [60, 65], which may point to potential differences between patient cohorts and/or ethnic groups.

The median plasma concentrations of ANGPTL4, TNF-α, PEDF, and EPO were not statistically different from their respective controls. Nevertheless, some variations were apparent. Although not reaching significance, EPO was high in the nAMD and PDR group where increased levels have been reported before [38, 66]. Both groups have a strong vascular phenotype as a result of hypoxic insults. As a classical hypoxia-regulated protein, increased EPO levels in these patients may thus reflect the hypoxic state of the ocular tissue that led to the vascular phenotype. This is also reflected in median levels of ANGPTL4, which are elevated in all groups compared to non-hypoxic controls (ERM). Elevated levels of ANGPTL4 in PDR in patients were already reported before and the factor is discussed as potential therapeutic target for patients suffering from diabetic retinopathy [24]. Elevated plasma levels of PEDF and TNF-α were demonstrated in PDR patients [67–69]. PEDF was also increased in nAMD when compared to dry AMD [70]. The authors also reported a positive correlation between VEGF and PEDF in the nAMD group. These data were not corroborated in our study.

Although significant changes in intraocular levels of the investigated factors have been reported for pathologies involving hypoxic conditions [71–76]**,** most changes in the circulation of our patient groups were only minor, the exception being PlGF. Why only some of the hypoxia-regulated factors that were found to be increased in the eye were elevated in plasma of patients while others were not is unclear. Possible explanations may include different stabilities of the proteins in ocular fluids and plasma, differences in the cell types producing the factors, differential secretion, or others. Additionally, differences in patient care, e.g., variability in the control of blood sugar levels, potential differences between ethnic groups, and the size of the cohorts tested may influence data outcome and explain differences to published data. It seems likely, however, that ocular concentrations of specific factors in the diseased eye do not necessarily correlate with factor concentrations in plasma. Therefore, our data suggest that the response to hypoxic events in retinal pathologies remains mostly local, even though many factors produced by such conditions are secreted proteins. The confinement of hypoxia-induced angiogenic factors to the local environment of the eye might thus help to prevent potentially dangerous systemic neo-angiogenesis in patients. This possibility needs to be taken into consideration when searching for circulating biomarkers for eye pathologies.

## Strengths/limitations

The strength of our study is that we used a protocol ensuring that all procedures were performed the same way in all patients. Furthermore, we studied and compared for the first time to our knowledge three different groups with the following retinal hypoxic diseases: nAMD, dry AMD, and PDR as well ERM as a non-hypoxic control group. This simultaneous analysis allows for a direct comparison of values measured.

However, the limitation of the study is that the number of the enrolled patients was very small and not equal for all groups, making it difficult to demonstrate comparability among the groups. Since only very few cytokine studies have been conducted so far, the availability of commercial kits was limited and their use needed some tweaking to work efficiently. Moreover, we restricted the exclusion criteria only to eye conditions without referring to any systemic diseases, which could also have an influence on plasma levels of the studied factors.

## Conclusions

Based on the current results, we hypothesize that local intraocular regulatory mechanisms regarding cytokine secretion under hypoxic conditions exist [50, 66] that are unrelated to systemic regulation. Trends in the different cytokines such as elevated levels of ANGPTL4 in the dry AMD group suggest new avenues for therapeutic targets in dry AMD. Since our study included only a small number of patients, further studies with larger patient groups are needed to verify a possible systemic expression of factors related to hypoxia-induced angiogenesis in the eye.
